# Antimicrobial Preservatives in Cyclodextrin-Containing Drug Formulations

**DOI:** 10.3390/pharmaceutics16121601

**Published:** 2024-12-16

**Authors:** Phatsawee Jansook, Hákon Hrafn Sigurdsson, Frédéric Pilotaz, Thorsteinn Loftsson

**Affiliations:** 1 Faculty of Pharmaceutical Sciences, Chulalongkorn University, 254 Payathai Road, Pathumwan, Bangkok 10330, Thailand; phatsawee.j@chula.ac.th; 2 Cyclodextrin Application and Nanotechnology-Based Delivery Systems Research Unit, Chulalongkorn University, Bangkok 10330, Thailand; 3 Faculty of Pharmaceutical Sciences, University of Iceland, Hofsvallagata 53, IS-107 Reykjavik, Iceland; hhs@hi.is; 4 Horus-Pharma, Département des Opérations Industrielles, 22, allée Camille-Muffat, bât. Inedi 5, 06200 Nice, France

**Keywords:** antimicrobial preservative, cyclodextrin, inactivation, antimicrobial efficacy enhancer

## Abstract

In general, antimicrobial preservatives are essential components of multidose pharmaceutical formulations to prevent microbial growth and contamination, many of which contain lipophilic and poorly water-soluble drugs in need of solubilizing excipients, such as cyclodextrins (CDs). However, CDs frequently reduce or even abolish the antimicrobial activities of commonly used pharmaceutical preservatives. The degree of inactivation depends on the CD complexation of the preservatives, which in turn depends on their chemical structure and physiochemical properties. In general, lipophilic preservatives are more likely to be inactivated; however, CDs are also known to inactivate highly water-soluble preservatives. In some drug formulations, preservative inactivation has been offset by including excipients that possess some antimicrobial activity on their own. In this review, we explain how CDs interact with some commonly used pharmaceutical preservatives and why some preservatives are more susceptible to CD inactivation than others are.

## 1. Introduction

In general, antimicrobial preservatives are essential components of multidose pharmaceutical formulations in aqueous solutions that prevent microbial growth and contamination. These preservatives must possess broad-spectrum antimicrobial activity and stability, be non-irritating and non-toxic, and be compatible with other formulation components and primary packaging materials. Owing to multiple interactions in aqueous formulations, only a fraction of a given preservative is active, emphasizing the importance of understanding the physicochemical properties of preservatives and the effects of formulation composition. At least 20 different antimicrobial preservatives can be found in marketed pharmaceutical products, in addition to various excipients that can increase their antibacterial activity, such as chelating agents, essential oils, alcohols, and fatty acids, which possess some antimicrobial activity on their own and can have additive or synergistic antimicrobial effects [1,2,3,4,5,6]. The formulation type can also affect the antimicrobial efficacy of preservatives. For example, o/w emulsions consist of a dispersed oil phase and a homogeneous aqueous phase. Microbial cells are mainly located in the aqueous phase, whereas preservatives are distributed between the two phases. Similarly, preservatives can bind to solid particles in pharmaceutical suspensions and to polymers in hydrogels. For a preservative to affect microbial growth, the concentration of the free preservative molecules in the aqueous phase must be sufficient. Water-soluble polymers, complexing agents, and surfactants can bind dissolved preservative molecules and render them inactive in aqueous solution. Furthermore, preservatives may be absorbed from aqueous media into plastics, rubber, and other elements of primary packaging materials, thereby reducing their activity. These incompatibilities have been described in many well-known pharmaceutical textbooks [7,8,9]. Although preservatives can prevent microbial contamination, they frequently cause other problems such as allergic reactions, irritation, and toxic side effects. Thus, it is recommended that the preservative concentration in pharmaceutical formulations should be maintained at levels just sufficient to comply with the compendial antimicrobial efficacy tests (e.g., the United States Pharmacopeia National Formulary (USP) Chapter < 51 > “Antimicrobial Effectiveness Testing” and the European Pharmacopeia (Ph. Eur.) Chapter 5.1.3 “Efficacy of Antimicrobial Preservation”). The tests are performed during drug formulation development and are harmonized with respect to how the tests are performed but differentiate between challenging microorganisms and acceptance criteria.

Cyclodextrins (CDs) reduce or abolish the antimicrobial activity of preservatives in aqueous pharmaceutical formulations. In general, CDs have a much greater effect than other common excipients [10,11,12,13,14,15]. Studies have also shown that by proper selection of CDs and adjusting their concentration, it is possible to minimize the effect of CDs on antimicrobial activity [16]. CDs can also enhance the efficacy of lipophilic antimicrobial agents [17,18,19,20,21,22,23], possibly by increasing their solubility and availability in aqueous formulations [24,25,26]. Thus, CDs can decrease or occasionally enhance the antimicrobial activity of preservatives and other antimicrobial compounds. In some cases, excipients such as propylene glycol and boric acid can be added to counteract CD inactivation of preservatives such as benzalkonium chloride (BAC) and other quaternary ammonium compounds [27,28]. Holm et al. developed a mathematical model to calculate the required concentrations of both neutral (e.g., parabens) and ionized (e.g., benzoate) preservatives in aqueous CD solutions containing poorly soluble drugs [14]. Holm’s model is based on the stability constants of the drug/CD (D/CD) and preservative/CD (P/CD) complexes and the minimum inhibitory concentration (MIC) of the preservative. This model can explain the rather complex interactions in pure aqueous solutions containing a drug, preservative, and CD and helps formulators estimate the required preservative surplus. However, the efficacy of the final preservative concentration in a given formulation should be verified by a compendial antimicrobial efficacy test. CD inactivation means that the formulation no longer complies with the compendial antimicrobial efficacy tests due to CD complexation of the preservative. The following is a review of how CDs affect the effectiveness of common antimicrobial preservatives in aqueous pharmaceutical formulations. The CDs found in marketed pharmaceutical products include α-cyclodextrin (αCD), β-cyclodextrin (βCD), 2-hydroxypropyl-β-cyclodextrin (HPβCD), sulfobutyl ether β-cyclodextrin sodium salt (SBEβCD), randomly methylated β-cyclodextrin (RMβCD), γ-cyclodextrin (γCD), and 2-hydroxypropyl-γ-cyclodextrin (HPγCD). All reported percentage concentrations are weight by volume (% *w*/*v*).

## 2. Classification of Preservatives and Their Physiochemical Properties

**Figure 1:**
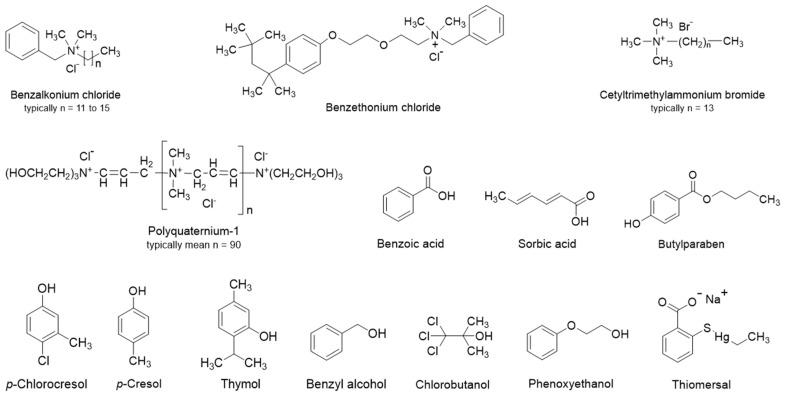
Structures of commonly used antimicrobial preservatives.

Pharmaceutical formulators generally prefer antimicrobial preservatives with compendial monographs, such as in Ph. Eur. or USP [5,29,30]. Examples of such preservatives are quaternary ammonium compounds such as benzalkonium chloride, cetrimonium bromide, and cetylpyridinium chloride; organic acids such as benzoic acid and sorbic acid, as well as their salts; parahydroxybenzoate esters (i.e., parabens) such as methylparaben, ethylparaben, propylparaben, and butylparaben; phenols such as chlorocresol, cresol, phenol, and thymol; and alcohols such as chlorobutanol, benzyl alcohol, and phenoxyethanol (Figure 1). The use of organomercurial preservatives (e.g., thiomersal) in pharmaceutical products is limited because of toxicity concerns [31]. The efficacy of an antimicrobial preservative in a given liquid pharmaceutical formulation is determined by the composition of the formulation and its pH, as well as the structure and physiochemical properties of the preservative. The physicochemical properties of common preservatives are listed in Table 1. Frequently, the antimicrobial activity of ionizable compounds is pH dependent. For example, phenols (pKa about 10) are inactive at pH values above 9, where they are ionized, and benzoic acid (pKa 4.2) has optimum antibacterial activity at pH values between 2.5 and 4.5 [30]. The tendency of preservatives to be absorbed into plastic containers and oil droplets in o/w emulsions increases with increasing lipophilicity. The antimicrobial activity of some preservatives, such as parabens, increases with increasing lipophilicity [32]. Quaternary ammonium compounds are inactivated by various polymers, anionic surfactants, and other anionic compounds via electrostatic interactions [30].

The number of hydrogen bond donors and acceptors in a preservative influences its ability to form bonds with surrounding molecules, including other pharmaceutical excipients [33,34]. In other words, the chemical structure and physiochemical properties of the preservatives determine the location and availability of the preservative molecules within the pharmaceutical formulation and the preservative concentration in contact with microbial contamination. Only a fraction of the dissolved preservative molecules can bind to microbes in aqueous pharmaceutical formulations to achieve the desired antimicrobial effect.

## 3. Preservative—Cyclodextrin Interactions

CDs can form inclusion and sometimes non-inclusion complexes with preservatives, and the extent of such complexation is determined by the structure and physicochemical properties of the preservative. Most frequently, the concentrations of antimicrobial preservatives in the formulations are low (below 0.1% *w*/*v*); thus, even low CD concentrations can affect the antimicrobial availability of preservatives. At low concentrations, CDs enhance the antimicrobial activity of lipophilic and poorly water-soluble preservatives. However, at high CD concentrations, CDs can reduce antimicrobial activity (see Section 4). The most common type of preservative/CD complex (P/CD) in aqueous formulations, especially in dilute solutions, is a 1:1 complex in which one preservative molecule forms a complex with one CD molecule:

(1) $$P+CD\overset{K_{1:1}}{↔}P/CD$$

Under such conditions, the concentration of free preservative in a saturated solution can be calculated from the complexation efficacy (CE):

(2) $$\mathrm{CE}=K_{1:1}\cdot S_{0}=\frac{\left[P/\mathrm{CD}\right]}{\left[\mathrm{CD}\right]}=\frac{\mathrm{Slope}}{1\;-\;\mathrm{Slope}}$$

(3) $$f_{\mathrm{free}\;P}=\frac{\left[P\right]}{{\left[P\right]}_{T}}=\frac{1}{{1+K}_{1:1}\cdot \left[CD\right]}$$

where K_1:1_ is the stability constant of the P/CD complex, S_0_ is the preservative solubility in the aqueous formulation when no CD is present, slope is the slope of the linear phase-solubility diagram (in moles/L), [P] is the concentration of the free preservative, [P/CD] is the concentration of the complex, f_free P_ is the fraction of free preservative in a CD solution, and [P]_T_ is the total preservative concentration (i.e., [P] + [P/CD]) [35]. Comparable equations can be derived for drugs (D). Table 2 shows examples of the stability constants and estimated CEs for some preservative/CD complexes. These values may vary depending on the experimental conditions and the excipients present in the complexation media.

**Figure 2:**
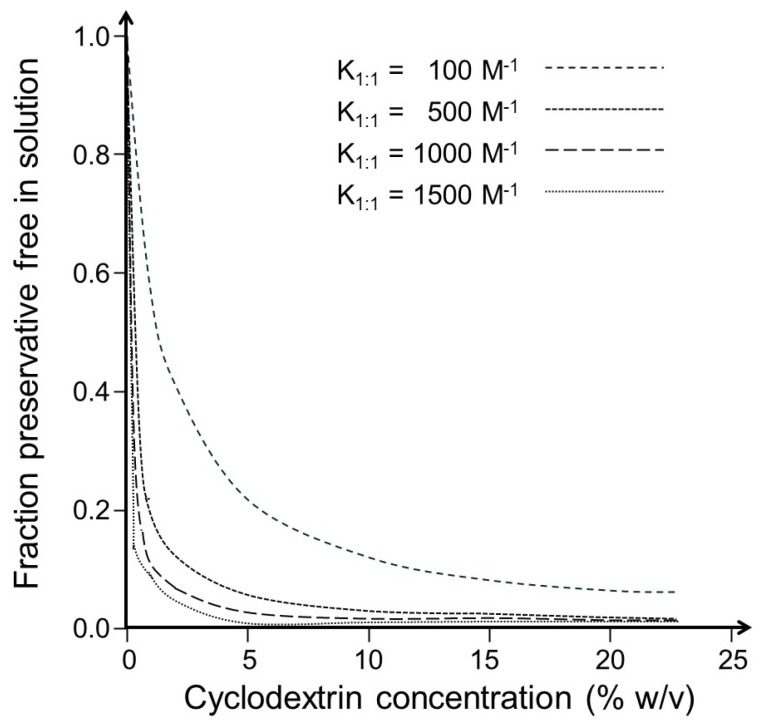
The figure shows how the fraction of free preservative in aqueous CD solution (f_free P_, Equation (3)) is affected by the value of the stability constant (K_1:1_) of the preservative/CD complex and the CD concentration. The molecular weight of CD was set at 1400 g/mol, and the values of K_1:1_ at 100, 500, 1000, and 1500 M^−1^.

In aqueous drug formulations, CD concentration is determined by the amount needed to dissolve a given amount of drug, and generally, a small excess (e.g., 10%) is included to prevent drug precipitation during manufacturing and storage. The high CD concentration needed to dissolve the drug and the low CE of the drug-CD complex, leading to a high concentration of free CD molecules, push the equilibrium towards the preservative-CD complex (Equation (1)), decreasing the fraction of free preservatives even further. Figure 2 shows the dependence of the free fraction of a preservative on the CD concentration and the value of K_1:1_ of the preservative-CD complex in a pure aqueous CD solution (Equation (3)). The fraction decreased with increasing CD concentration and increasing value of K_1:1_. In other words, the degree of CD inactivation of a preservative is determined by the K_1:1_ of the preservative-CD complex and the CD concentration (Equation (3)).

The drug-to-CD molar ratio (Equation (4)) in the CD solution is determined as follows [35]:

(4) $$\mathrm{D:CD}\;\mathrm{molar}\;\mathrm{ratio}\;\mathrm{in}\;\mathrm{drug}\;\mathrm{saturated}\;\mathrm{CD}\;\mathrm{solution}=1:\frac{\mathrm{CE}+1}{\mathrm{CE}}$$

**Figure 3:**
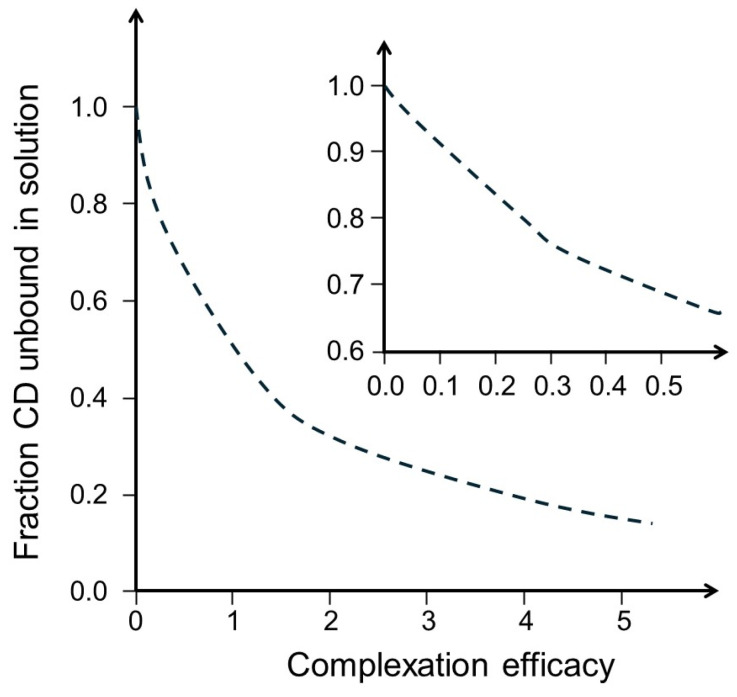
Fraction of cyclodextrin unbound in aqueous solution saturated with a drug as a function of the complexation efficacy assuming formation of 1:1 D:CD complexes, based on data from Table 3.

The fraction of unbound CD in the solution can be calculated from the D:CD molar ratio. The data in Table 3 were used to draw Figure 3, which shows that when the CE was 0.1, about 90% of the dissolved CD was unbound and 80% when the CE was 0.25. Even when the CE is relatively high, as in the case of hydrocortisone in aqueous HPβCD solution, more than one-third of the CD molecules are unbound and can form a complex with the preservative in a CD solution saturated with the drug. The fraction of unbound CD is much higher because excess CD is required to prevent drug precipitation during storage and handling. Thus, the concentration of free (i.e., unbound) CD molecules is always relatively high in aqueous CD-containing drug formulations. These factors may influence the optimal preservative concentration in multi-dose CD-based formulations needed to achieve sufficient antimicrobial efficacy without causing adverse effects.

## 4. Studies of Antimicrobial Efficacy in Aqueous CD Solutions

Numerous reports have described the effect of CD concentration on the antimicrobial activity of preservatives in aqueous solutions (Table 4). Generally, antimicrobial inactivation increases with increasing CD concentrations. However, the degree of inactivation depends on the fraction of free preservatives in the aqueous solution (Equation (3)). For example, benzalkonium chloride (BAC) is a potent preservative with good solubility in water but a high K_1:1_ (>10^3^ M^−^^1^, Table 1 and Table 2). Thus, BAC was not affected by low CD concentrations but displayed significant inactivation at higher CD concentrations. Notably, water-soluble preservatives such as BAC still form inactive water-soluble CD complexes in aqueous solutions. The antimicrobial efficacy of benzoic acid depends on ionization; the unionized form has higher antimicrobial activity; however, the unionized form is more lipophilic and has a higher affinity for the CD cavity (i.e., higher K_1:1_) [14]. Methyl paraben (solubility 5.5 mg/mL and effective concentration 0.1 to 0.4 mg/mL) has relatively high affinity for βCD and its derivatives (K_1:1_ from approx. 800 to 1500 M^−^^1^) and thus is inactivated by relatively low βCD concentrations (Table 1 and Table 2). At physiological pH, thiomersal is very hydrophilic (LogD_7_—1.86, solubility approximately 1000 mg/mL at pH 7), and although it has significant affinity for HPβCD in its unionized form, HPβCD does not have much effect on its antimicrobial efficacy at physiological pH, where thiomersal is fully ionized. Although CDs are able to form complexes with both drugs and excipients, drugs generally do not have much effect on CD inactivation of preservatives, partly due to the presence of excess CD needed to prevent drug precipitation during manufacturing and storage of the product and partly due to the low preservative concentrations.

## 5. Examples of Marketed Products

Recently, Puskás et al. published a list of 130 approved pharmaceutical products formulated with parent CDs (i.e., αCD, βCD, and γCD) or their derivatives (e.g., HPβCD, SBEβCD, and RMβCD), approximately 50 of which are aqueous solutions [55]. Only a few are marketed in multidose containers, some of which contain antimicrobial preservatives. The exact composition of these formulations is, in most cases, not readily available; however, in some cases, the relevant patent literature reports nearly identical formulations. For example, Indocollyre^®^ eye drops (Laboratoire Chauvin, Bausch & Lomb, Aubenas, France) are an aqueous solution containing 0.1% indomethacin, HPβCD, arginine, and thiomersal (i.e., mercurothiolate sodium). According to a European patent (EP0761217A1, Assignee: Laboratoires Chauvin), the composition may contain as much as 10% (*w*/*v*) HPβCD [56]. However, thiomersal is a potent and highly hydrophilic antimicrobial preservative with high solubility in water (Table 1) and low affinity for HPβCD in its ionized form, and therefore, it provides acceptable antimicrobial preservation in this eye drop formulation. As Indocollyre^®^ is approved in the EU, its formulation passes the Eur. Ph. antimicrobial efficacy test. The Vitrakvi^®^ oral solution (Bayer, Reading, UK; Loxo Oncology, Stamford, CT, USA) contained larotrectinib (2%) in a vehicle composed of sucrose (29.5%), HPβCD, glycerol, sorbitol (2.2%), sodium citrate, sodium phosphate, citric acid, propylene glycol (0.12%), potassium sorbate, methylparaben (0.02%), and flavoring agents in water. Patent literature (US11191766B2, Assignee: Loxo Oncology, Stamford, CT, USA) indicates that the HPβCD concentration is close to 15% (*w*/*v*) [57]. Methylparaben has a significant affinity for HPβCD (high K_1:1_ value; Table 2); thus, HPβCD reduces its antimicrobial efficacy. In contrast, 29.5% sucrose and 15% HPβCD caused the Vitrakvi^®^ oral solution to become hyperosmotic. Preservation is achieved through hyperosmosis, the antibacterial activity of methylparaben, and possibly by the antimicrobial effects of citric acid and propylene glycol. Pataday^®^ extra-strength eye drops (Alcon, Fort Worth, TX, USA) are an aqueous solution containing 0.776% olopatadine hydrochloride (equivalent to 7 mg/mL olopatadine), povidone (4.0%), HPγCD (1.5%), polyethylene glycol 400 (4.0%), hydroxypropyl methylcellulose (0.4%), boric acid (0.3%), mannitol (0.2%), benzalkonium chloride (0.015%), and hydrochloric acid/sodium hydroxide (q.s. pH 7.2) (see https://www.accessdata.fda.gov/scripts/cdrh/cfdocs/cfrl/rl.cfm (accessed on 11 December 2024); Appl. No. 0206276Orig1s005; Approval Date: 13 July 2020). The low concentration of HPγCD most likely results in some BAC inactivation. However, the combination of boric acid and mannitol possesses antibacterial activity and provides buffering at approximately physiological pH [58]. Thus, acceptable microbial preservation of Pataday^®^ eye drops was achieved by combining BAC with boric acid and mannitol. Clorocil^®^ eye drops (Laboratório Edol, Linda-a-Velha, Portugal) containing chloramphenicol (0.8%) in a vehicle containing boric acid, borax, BAC (0.01%), dimethyl-β-cyclodextrin (CD), which is closely related to RMβCD, and sodium chloride in purified water. Chloramphenicol is a broad-spectrum antibiotic, although it is not effective against, for example, *Pseudomonas aeruginosa*. Thus, the preservative used in Clorocil^®^ eye drops consists of the active ingredient and BAC.

The slope of the linear phase-solubility diagram of diclofenac in aqueous HPγCD solutions is greater than unity, indicating that the stoichiometry of the diclofenac/HPγCD complex is first-order with respect to HPγCD but second or higher order with respect to diclofenac [59]. Thus, the CE and f_unbound_ CD cannot be calculated as described above. According to the packaging, Voltaren^®^ Ophtha CD (Novartis, Basel, Switzerland) contains diclofenac sodium (0.1%), benzalkonium chloride (0.005%), disodium edetate (EDTA), HPγCD, hydrochloric acid, propylene glycol, trometamol, tyloxapol, and water for injection (WFI). However, according to a Novartis patent application (WO1997010805A1; Applicant: Novartis AG, Basel, Switzerland), the composition may be as follows: diclofenac sodium (0.1%), benzalkonium chloride (0.005%), disodium edetate (0.1%), HPγCD (2.0%), hydrochloric acid (q.s. pH 7.96), propylene glycol (1.9%), trometamol (0.1%), tyloxapol USP (0.1%), and water for injections [27]. Sente et al. published a detailed description of the formulation development of Voltaren^®^ Ophtha CD eye drops, stating that HPγCD does not form a complex with BAC and, thus, does not inactivate its antimicrobial effect at this low HPγCD concentration [60]. However, it must be noted that BAC does form complexes with both γCD and HPγCD, but their K_1:1_ values are much lower than that of βCD and its derivatives [61]. According to the authors, propylene glycol acts as an isotonic agent in addition to supporting the efficacy of BAC in the presence of HPγCD, and tyloxapol counteracts the incompatibility between positively charged BAC and negatively charged diclofenac. Disodium edetate (EDTA) is an antimicrobial efficacy enhancer, and trometamol (synonyms: tromethamine and Tris) forms a Tris-EDTA buffer that can also act as a complexing agent and solubilizer. The eye drops passed the Eur. Ph. antimicrobial efficacy test [60].

## 6. Examples from the Patent Literature

The patent literature contains some examples of preserved aqueous solutions containing CDs, and preservation is frequently obtained by combining several antimicrobial excipients. The antimicrobial preservative Purite^®^ (Bio-Cide International Inc., Norman, OK, USA) is an aqueous solution of an oxychloro complex in an equilibrium mixture of oxychloro species, predominantly chlorite, chlorate, and chlorine dioxide. A patent (US 6,933,289 B2) described prostaglandin eye drops containing 0.03% bimatoprost in an aqueous pH 7.3 solution containing HPβCD (1.0%), carboxymethylcellulose (1.0%), boric acid (0.60%), sodium borate (0.045%), sodium chloride, potassium chloride, calcium chloride, magnesium chloride, and Purite^®^ (0.01%) [62]. Purite^®^ is a preservative, but boric acid/borate possesses some antibacterial effects and, thus, enhances antimicrobial efficacy. One patent (US 5,985,310) contains examples of aqueous 0.5% betaxolol eye drops containing HPβCD (7.5%), boric acid (0.5%), sodium chloride (0.3%), EDTA (0.01%), polyquaternium-1 (0.01%), and sodium hydroxide/hydrochloric acid at pH 7.0 [28]. The preservative is a combination of polyquaternium-1 and boric acid as well as the antimicrobial enhancer EDTA. CDs, especially SBEβCD, are known to enhance the antimicrobial activity of various compounds [63,64,65]. At sufficiently high concentrations, aqueous SBEβCD solutions can be characterized as self-preserved (US 10,463,677 B2 and US 2005/0250738 A1) [66,67]. A patent application (WO2023148231) describes an aqueous eye drop suspension containing 1.5% dexamethasone, 14% γ-CD, 2.5% poloxamer, 0.47% sodium thiosulfate, 0.10% EDTA, and 0.10% sodium chloride in purified water at pH 4.0 [61]. Even at this high γCD concentration, the eye drops passed the USP antimicrobial efficacy test. Sorbic acid has a very low affinity for CDs; thus, CDs generally have little effect on the antimicrobial activity of sorbic acid (see Table 2 and ref. [61]). However, sorbic acid and sorbate are not highly effective antimicrobial agents, and their antimicrobial activity is strongly influenced by the pH; thus, this formulation failed the Ph.Eur antimicrobial efficacy test.

## 7. Conclusions

CDs influence antimicrobial activities by reducing or inhibiting the effectiveness of commonly used preservatives in pharmaceutical aqueous solutions. The chemical structure and physicochemical properties of preservatives play a crucial role in determining their affinity for CDs and susceptibility to inactivation. Preservatives with high hydrophilicity exhibit lower affinity for CDs and are less prone to inactivation, while water-soluble preservatives with lipophilic moieties may form inclusion complexes with CDs, leading to inactivation. In some cases, excipients with inherent antimicrobial properties, such as EDTA, boric acid, borax, and zinc ions, can enhance the preservation efficacy in aqueous CD formulations. Thus, the development of multiple-dose CD-based formulations should consider the physicochemical properties of preservatives, as well as the types and concentrations of CDs used, which affect the availability of free preservatives in the formulation. Furthermore, the effectiveness of the final preservative concentration in a CD-containing formulation should be verified using a compendial antimicrobial efficacy test.

## Figures and Tables

**Table 1:** Some antimicrobial preservatives that are used in pharmaceutical products. Data collected from Chemical Abstracts Services (scifinder-n.cas.org, accessed on 11 December 2024) and Pubchem (pubchem.ncbi.nlm.nih.gov, accessed on 11 December 2024). It should be noted that for non-ionizable drugs LogD is equal to LogP throughout the pH range.

Preservative	Molecular Weight (g/mol)	pKa	H-Bonds		LogD_4_	LogD_7_	Solubility (mg/mL) **	
			Donors	Acceptors			pH 4	pH 7
Benzethonium chloride	448.08	-	0	3	-	4.0	>10	>10
Benzoic acid	122.12	4.20	1	2	1.35	−1.08	8.8	1000
Benzyl alcohol	108.14	-	1	1	1.06	1.06	47	47
Benzyldodecyldimethylammonium chloride *	339.99	-	0	1	2.63	2.63	866	866
Butyl paraben	194.23	8.22	1	3	3.41	3.38	0.50	0.54
Chlorobutanol	177.46	-	1	1	1.73	1.73	10	10
Chlorohexidine	505.45	11.51	10	10	1.56	1.58	1.1	1.0
m-Cresol	108.14	10.07	1	1	2.04	2.04	23	23
Diazolidinyl urea	278.22	11.22	5	11	−5.40	−5.40	999	999
Imidazolidinyl urea	388.29	7.41	8	16	−4.93	−5.02	0.002	0.002
Isobutyl paraben	194.23	8.17	1	3	3.25	3.23	0.56	0.60
Methyl paraben	152.15	8.31	1	3	1.88	1.86	5.5	5.6
Phenol	94.11	9.86	1	1	1.54	1.54	96	96
Phenoxyethanol	138.16	-	1	2	1.25	1.25	17	17
Polyquaternium-1	>800	-	6	≥8	−9.90	−9.90	***	***
Propyl paraben	180.20	8.23	1	3	2.90	2.88	1.1	1.2
Quaternium-15	251.16	3.7	0	4	-	−0.1	-	1000
Sorbic acid	112.13	4.60	1	2	1.17	−1.12	11	1000
Thiomersal	404.82	3.62	0	3	-	−1.88	-	1000

* One of the main components of benzalkonium chloride (BAC), which is a mixture of alkyl dimethyl benzyl ammonium chlorides. ** Calculated solubility that may divide from experimental solubility. *** Low molecular weight polyquaternium-1 (810 g/mol) is soluble in water, but the solubility decreases with increasing molecular weight. High-molecular-weight polyquaternium-1 (30,000 g/mol) is only slightly soluble in water.

**Table 2:** Effective concentrations in aqueous solutions, stability constants (K_1:1_), and estimated complexation efficacy (CE) (Equation (2)) in aqueous cyclodextrin solution saturated with the preservative at 20 to 30 °C.

Preservative	Effective Conc. (% *w*/*v*)	Cyclodextrin	K_1:1_ (M^−1^)	CE	Ref.
Benzalkonium chloride	0.004–0.02	βCD	1400	3500	[36]
Benzoic acid, unionized	0.1–0.2	βCD	678	30	[14]
		RMβCD	1013	44	[14]
		HPβCD	536	36	[14]
		SBEβCD	924	41	[14]
Benzyl alcohol	0.5–5	αCD	22	9.5	[37]
		βCD	50	22	[37]
Butyl paraben	0.02–0.4	αCD	701	0.38	[38]
		HPαCD	323	0.18	[38]
		βCD	4582	2.5	[38]
		HPβCD	16,240	9.0	[38]
Chlorohexidine	0.1–0.2	βCD	268	0.58	[39]
m-Cresol	0.15–0.3	βCD	95	20	[40]
Ethyl paraben	0.1–0.3	αCD	193	0.84	[38]
		HPαCD	149	0.65	[38]
		βCD	1709	7.46	[38]
Methyl paraben	0.01–0.4	HPαCD	67	1.3	[38]
		βCD	772	27	[14]
		RMβCD	1453	52	[14]
		HPβCD	1128	28	[14]
		SBEβCD	1519	55	[14]
Phenol	0.2–0.5	βCD	129	129	[41]
Phenoxyethanol	0.25–0.5	HPβCD	100	12	[13]
Propyl paraben	0.005–0.1	αCD	240	0.42	[38]
		HPαCD	230	0.39	[38]
		βCD	1548	9.3	[14]
		RMβCD	3544	21	[14]
		HPβCD	2360	16	[14]
		SBEβCD	3165	19	[14]
Sorbic acid, unionized	0.05–0.5	αCD	[119] ^1^	[2.2] ^1^	[42]
		HPβCD	[42] ^1^	[0.76] ^1^	[42]
Thiomersal	0.001–0.1	HPβCD	1916	19	[13]

^1^ Estimated value from a phase-solubility diagram.

**Table 3:** Molecular weight (MW), solubility (S_0_) of the unionized drug in pure water, approximate complexation efficacy (CE), the drug:CD molar ratio (Equation (4)), and fraction of unbound CD (f_unbound_) in aqueous cyclodextrin (CD) solution saturated with the drug at ambient temperature assuming formation of 1:1 D/CD complexes.

Drug	MW (g/mol)	S_0_ (M)	Cyclodextrin	CE	D:CD Molar Ratio	f_unbound_	Ref.
Acetazolamide (pKa 7.4)	222.25	0.003	HPβCD	0.246	1:5	0.80	[43]
			RMβCD	0.566	1:3	0.67	[44]
			HPγCD	0.021	1:50	0.98	[44]
Amphotericin B (pKa 5.7, 10.0) ^1^	924.09	0.000002	αCD	0.002	1:500	1.0	[45]
			βCD	0.001	1:1000	1.0	[45]
			γCD	0.069	1:16	0.94	[45]
			HPγCD	0.039	1:27	0.96	[45]
Axitinib (pKa 4.3) ^2^	386.47	0.000001	γCD	0.0002	1:5000	1.00	[46]
Brinzolamide (pKa 5.9, 8.4)	383.51	0.001	γCD	0.02	1:50	0.98	[47]
			HPγCD	0.03	1:35	0.97	[47]
Candesartan cilexetil (pKa 3.5, 5.9) ^3^	610.66	0.00001	γCD	0.0012	1:835	1.0	[48]
Celecoxib (pKa 9.6)	381.37	0.000003	αCD	0.0001	1:10,000	1.00	[49]
			βCD	0.0022	1:500	1.00	[49]
			γCD	0.0004	1:2500	1.00	[49]
			HPβCD	0.0075	1:135	0.99	[49]
			RMβCD	0.0089	1:113	0.99	[49]
Cyclosporin A	1202.61	0.00001	HPβCD	0.004	1:250	1.00	[43]
Dexamethasone	392.46	0.0004	HPβCD	0.326	1:4	0.75	[43]
Dovitinib (pKa 7.7) ^2^	392.43	0.00002	γCD	0.011	1:92	0.99	[46]
Fenofibrate	360.83	0.00001	αCD	0.20	1:6	0.83	[50]
			βCD	1.85	1:1.5	0.33	[50]
			γCD	0.21	1:6	0.83	[50]
			SBEβCD	0.63	1:3	0.67	[50]
			HPβCD	2.62	1:1.4	0.29	[50]
			RMβCD	4.54	1:1.2	0.17	[50]
Fluorometholone	376.46	0.00008	SBEβCD	1.91	1:1.5	0.33	[51]
			HPγCD	0.467	1:3	0.67	[51]
Hydrocortisone	362.46	0.001	HPβCD	2.00	1:1.5	0.33	[43]
Irbesartan (pKa 4.1, 7.4) ^3^	428.53	0.00001	γCD	0.289	1:5	0.80	[48]
Methazolamide (pKa 7.3)	236.26	0.004	γCD	0.04	1:26	0.96	[47]
			HPγCD	0.05	1:21	0.95	[47]
Naproxen (pKa 4.84) ^2^	230.26	0.0056	HPβCD	1.29	1:1.8	0.44	[52]
Triamcinolone acetonide	434.50	0.0003	HPβCD	0.063	1:17	0.94	[43]
Voriconazole (pKa 1.7)	349.31	0.002	αCD	0.066	1:17	0.94	[53]
			βCD	0.658	1:3	0.67	[53]
			RMβCD	0.545	1:3	0.67	[53]
			HPβCD	0.668	1:3	0.67	[53]

^1^ pH 5.5. ^2^ Phosphate buffer, pH 7.5. ^3^ pH 6.4 to 6.8.

**Table 4:** Effects of CDs on the antimicrobial efficacy of some preservatives.

Preservative	Comment	Ref.
Benzalkonium chloride (BAC)	In aqueous solution, the preservative efficacy was not affected by 0.5% HPβCD but 5% HPβCD had a significant effect.	[10]
	HPβCD and SBEβCD reduced the antimicrobial efficacy of BAC, both in the presence and absence of 0.1% EDTA, and the presence of the competing drug (0.1% fluorometholone) had no effect. Aqueous eye drops containing 0.1% fluorometholone, 5% HPβCD, 0.02% BAC, and 0.1% EDTA ^1^ passed the USP antimicrobial efficacy test.	[16]
Benzethonium chloride	1.1% (10 mM) βCD results in an almost 1000-fold increase in the MIC.	[12]
Benzoic acid (pKa 4.2)	Aqueous 1% citric acid ^1^ solution containing 5% HPβCD passes the European Pharmacopoeia antimicrobial efficacy test at benzoic acid concentrations ≥0.15% at pH 4.0 and ≥0.36% at pH 5.0.	[14]
Chlorobutanol	In aqueous solution, the preservative efficacy was not affected by 0.5% HPβCD but 5% HPβCD had significant effect.	[10]
m-Cresol	An exponential increase in m-cresol inactivation was observed with rising HPβCD concentration.	[15]
Methyl paraben	Aqueous 1% citric acid ^1^ solution, pH 5.0, containing 5% HPβCD passes the USP and Eur. Ph. antimicrobial efficacy test at preservative concentrations ≥ 0.46%.	[14]
	HPβCD and SBEβCD reduced antimicrobial efficacy, both in the presence and absence of 0.1% EDTA ^1^, and the presence of the competing drug (0.1% fluorometholone) had no effect.	[16]
Thiomersal	Thimerosal is water soluble and a very potent antimicrobial preservative. The antimicrobial activity of thimerosal is not inhibited by 4.5% HPβCD.	[15,54]

^1^ Citric acid and ethylenediaminetetraacetic acid (EDTA) are antimicrobial enhancers.

## Data Availability

This article is a review of the existing literature, and it does not involve the generation or analysis of new datasets. All data referenced in this review are available from the cited original publications, which are publicly accessible through the respective journals or databases.
